# A Portable Fuzzy Driver Drowsiness Estimation System

**DOI:** 10.3390/s20154093

**Published:** 2020-07-23

**Authors:** Alimed Celecia, Karla Figueiredo, Marley Vellasco, René González

**Affiliations:** 1 Electrical Engineering Department, PUC-Rio, Rio de Janeiro 22451900, Brazil; acelecia@aluno.puc-rio.br; 2 Department of Informatics and Computer Science, Institute of Mathematics and Statistics, State University of Rio de Janeiro (UERJ), Rio de Janeiro 20550-900, Brazil; 3 Research & Development Department, Solinftec, Araçatuba 16013337, Brazil; rene.hernandez@solinftec.com

**Keywords:** drowsiness detection, drowsiness measures, fuzzy inference system, Raspberry Pi, embedded hardware, eyes closing detection

## Abstract

The adequate automatic detection of driver fatigue is a very valuable approach for the prevention of traffic accidents. Devices that can determine drowsiness conditions accurately must inherently be portable, adaptable to different vehicles and drivers, and robust to conditions such as illumination changes or visual occlusion. With the advent of a new generation of computationally powerful embedded systems such as the Raspberry Pi, a new category of real-time and low-cost portable drowsiness detection systems could become standard tools. Usually, the proposed solutions using this platform are limited to the definition of thresholds for some defined drowsiness indicator or the application of computationally expensive classification models that limits their use in real-time. In this research, we propose the development of a new portable, low-cost, accurate, and robust drowsiness recognition device. The proposed device combines complementary drowsiness measures derived from a temporal window of eyes (PERCLOS, ECD) and mouth (AOT) states through a fuzzy inference system deployed in a Raspberry Pi with the capability of real-time response. The system provides three degrees of drowsiness (Low-Normal State, Medium-Drowsy State, and High-Severe Drowsiness State), and was assessed in terms of its computational performance and efficiency, resulting in a significant accuracy of 95.5% in state recognition that demonstrates the feasibility of the approach.

## 1. Introduction

Driver fatigue and drowsiness constitute one of the leading causes of traffic accidents, being involved in 9.5% of crashes in the US [1] and 6% of fatal accidents in Brazil [2]. To alleviate these figures, authorities, research groups, and automobile manufacturers have concentrated their efforts on developing awareness campaigns, promoting the implementation and use of rest stops, and developing automatic devices that assist drivers by detecting fatigue or drowsiness [3]. Particularly, automatic devices have shown promising capabilities [4,5], offering alternative solutions to alert drivers depending on the data obtained from multiple types of sensors.

There are three categories of drowsiness detection systems, based on the measures of these sensors [4]: Vehicle-Based, physiological, and behavioral. Vehicle-based measures rely on monitoring car parts or sensors (e.g., the steering wheel, pedals, or external cameras) to infer drivers’ level of fatigue or drowsiness based on driving habit modifications (e.g., a variation in steering wheel micro-corrections) or abnormal behavior (crossing lanes or leaving the road). Physiological measures include electroencephalography (EEG), electrooculography (EoG), electrocardiography (ECG), and electromyography (EMG) signals, and systems detect their deviation from the characteristics of the subject’s standard signals, and then analyze if the new state is related to drowsiness. Behavioral measures are mostly related to monitoring the driver’s face, focusing on facial features like eyes, brows, mouth, head inclination, or facial expression to determine the drowsiness level.

All three referred categories have some limitations that must be considered when developing a drowsiness detection device [4,5,6]. Vehicle-based measurements are associated with vehicle types, and are profoundly affected by many factors, such as: the driver’s habits, abilities, and experience; road characteristics (quality, marking status, lighting, geometry); and climate conditions. On the other hand, physiological measures are intrusive systems that demand that the user be connected to electrodes, which in turn are connected to some electronic processing device (even with the advances in wireless devices, the electrode would have to be on the subject). This setup involves noise and artefacts that can deteriorate the signal quality and therefore decrease the drowsiness detection accuracy. Additionally, EEG signals present a high variability among different subjects, as well as among measures from the same subject over time [7], demanding a very robust signal processing model to maintain the accuracy in continuous time. Limitations of the behavioral measures are strictly related to the sensors employed (i.e., camera(s)), which are affected by ambient lightning and vision obstruction (specifically when the subject uses glasses).

Among these different methods, behavioral measures are associated with lower cost, and are non-invasive, adaptable to any vehicle, and independent of road conditions. Therefore, a versatile and fully portable device which can function in real-time with an affordable price should be based on those measures. Drowsiness detection systems that employ these strategies have shown outstanding performance in experiments in both controlled laboratory and real conditions [8,9,10], given the limitations that behavioral measures can present (related to the sensors).

Usually, systems based on behavioral measures comprise a recording device for driver image acquisition, face recognition and identification of patterns indicating drowsiness, and an output signal of alarm. To ensure accurate performance, the most critical component is the hardware that processes the images and generates the alarm. Due to the high computing power demanded by computer vision algorithms, most of the portable drowsiness detection systems reported in the literature [5,8,9,10,11,12] are computer-based, employing a laptop for data processing. As a result, the cost of these systems is elevated, and their application is limited to specific scenarios, not being adaptable to different vehicle conditions.

Alternative processing hardware employed in these applications are smartphones [13,14,15,16,17,18] and semi-custom hardware devices [19,20,21]. In smartphone applications, the camera of the phone records the image of the subject, and drowsiness indicators are computed to generate the alarm signal. Given the high computing power and storage space of smartphone devices, these applications are capable of running, in real time (from 10 to 14 frames per second), complex algorithms like ensemble of classifiers [14] or deep neural networks [16,18] for face and drowsiness indicators recognition. On the other hand, due to hardware limitations, semi-custom hardware devices like field-programmable gate arrays (FPGAs) [19] and digital signal processors (DSPs) [20,21] are limited to methods which are less computationally expensive. In the case of one more complex drowsiness detection methodology [21], the response of the system was able to process at a reduced speed of only three frames per second (fps).

Given recent advances in the processing capacity of embedded hardware and single-board computers like the Raspberry Pi [22], Nvidia Jetson Nano [23], Asus Tinker Board [24], or Rock64 [25], such powerful hardware can represent a new step in the establishment of a low-cost drowsiness detection standard tool for driver safety maintenance. In particular, Raspberry Pi single-board computers are low-cost devices with impressive computing power, and have recently been applied in drowsiness detection systems [26,27,28,29,30]. In [26,27,28], the drowsy state was recognized, establishing a threshold for individual drowsiness indicators like percentage of eyelid closure (PERCLOS) [31] or detecting eye closure for a predefined time, which can limit the performance of the model in case of variations in the blinking frequency [8] or eye closure misclassifications. On the other hand, the application of convolutional neural networks in these devices is explored in [29,30], and due to the high computing capacity demanded by these algorithms, it was acknowledged that the response in real-time was limited, reporting a frame rate of 2.15 fps in [30]. This limitation led to an enhanced server-based processing model in a mobile phone device, which achieved 4.47 fps. From these results, authors in [26,27] tackled the limitations related to the variation of ambient lightning and vision obstruction, based on the addition of an infrared (IR) illuminator and camera system.

Therefore, to design a low-cost portable device applicable to any vehicle, it is necessary to overcome the limitations of the sensors employed in image acquisition. Additionally, it is necessary to develop a processing model with a balanced demand of computing capacity, robustness to facial features state misclassifications, and adaptable to characteristics from different subjects. In this sense, the combination of varying drowsiness indicators (including various facial features) such as PERCLOS, eye closing duration (ECD) [8], percentage of mouth yawn [9], and average mouth opening time (AOT) [10] can improve the robustness of the system. The use of multiple indicators overcomes the limitations of partially losing one of the facial features in the image, and of erroneous values from individual measures.

One of the suitable algorithms for the combination of these different modalities is a fuzzy inference system (FIS). Fuzzy inference systems are computationally inexpensive interpretable models which can consider various drowsiness indicators without establishing strict thresholds. These advantages were exploited in [32,33] for computer-based devices which evaluated only indicators extracted from the eyes region (PERCLOS, eyelid distance changes, eye closure rate, or ECD) to recognize if the driver was drowsy without addressing the problem of changes in the ambient illumination.

The main objective of this work was therefore to develop a new portable, low-cost, accurate, and robust drowsiness recognition device which incorporates complementary measures from eyes (PERCLOS, ECD) and mouth (AOT). The proposed equipment is based on a fuzzy inference system and deployed in a Raspberry Pi complemented with a NoIR camera and illumination system, which limits the influence of variations in ambient illumination and vision obstruction in drivers wearing glasses. The resultant device should be adaptable to any vehicle, indicating the drowsiness level of the user in three states: Normal State, Drowsy State, and Severe Drowsiness State.

The remainder of this paper is organized as follows: Section 2 describes the materials and methods employed to develop the hardware, software and processing algorithms; Section 3 describes the results obtained from testing the proposed device and associated algorithms under different conditions; finally, Section 4 provides the conclusions of the work.

## 2. Materials and Methods

**Figure 1:**
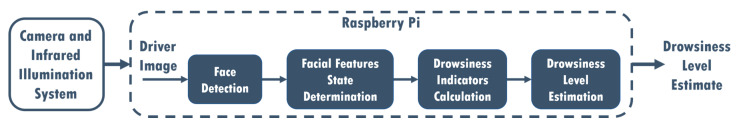
Portable Fuzzy Drowsiness Estimation System (PFDESys).

The proposed Portable Fuzzy Drowsiness Estimation System (PFDESys) is defined as observed in Figure 1.

The PFDESys is composed of five functional blocks: (i) a camera and infrared illumination system for the acquisition of the driver image; (ii) a face detection module to define the possible area in which the driver’s face is situated in the image; (iii) a module to determine the position of the facial features (eyes and mouth) and their states (closed or open); (iv) drowsiness indicators calculation module (PERCLOS, ECD, and AOT); and the driver drowsiness level estimation. The Raspberry Pi is the base hardware to run all algorithms defined for each functional block. The following subsections detail the methods applied in each processing block, finishing with a subsection that describes the hardware of the device.

### 2.1. Face Detection

Face detection is the first step when processing each driver’s image. In this work, we investigate two approaches: a cascade classifier that employs Haar-like filter features as proposed in [34], and a linear support vector machine (SVM) with histogram of oriented gradients (HOG) features, as proposed in [35]. Those approaches were chosen due to their reduced computational cost compared to deep learning approaches and proved accuracy.

The model proposed in [34] detects faces in an image with high velocity and accuracy, which is why it has been extensively utilized in the image processing community, extending its use to object detection applications. The algorithm defines a set of features derived from Haar filters that are applied to the image on a defined scale. The algorithm provides a high-dimensional set of features that is combined with an Adaboost model [36] to select a reduced number of those features and train a cascade of successive linear classifiers that best separate faces from no-faces in the images.

The second approach for face detection evaluated in this work is based on a feature set known as HOG features [35]. This feature extraction algorithm firstly divides the image into a grid of small cells, and then computes, for each image cell, the histogram of the orientation of the gradients weighted by their magnitude. Each cell histogram is stacked in a vector of features of high dimensionality after normalizing the values over a measure of the spatial energy on more extensive regions of the image. Face detection windows overlap with grids of HOG descriptors, providing a feature vector that can be employed with any classifiers. In this case, a linear SVM classifier was used.

### 2.2. Facial Features State Determination

**Figure 2:**
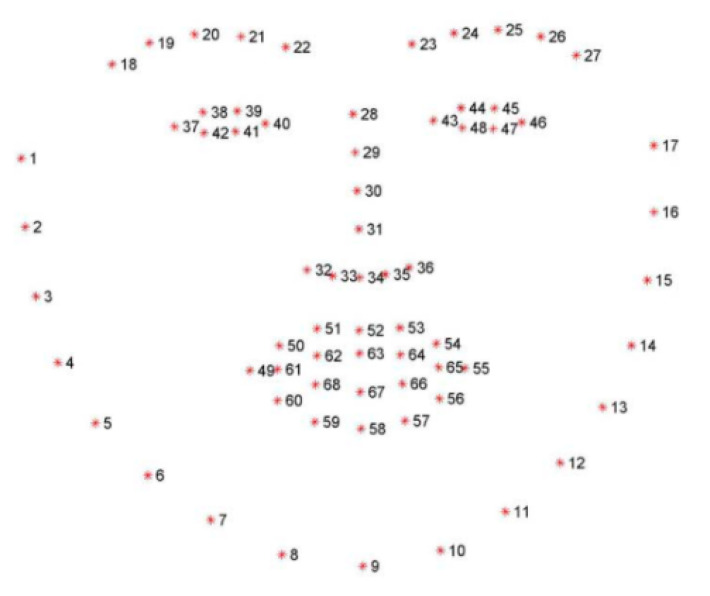
Facial landmarks included in the 300-W dataset.

After obtaining the possible region of the frame in which the face is situated, the algorithm reported in [37] is employed to determine the positions of facial landmarks. Facial landmarks describe main facial elements such as nose, eyes, eyebrows, and mouth, among others, through a series of marks as illustrated in Figure 2. The algorithm consists of a cascade of regression trees in which each regressor updates the estimated landmark positions employing the previous value and the intensity of a sparse set of pixels selected from the original estimate. The ensemble of regression trees is trained by gradient boosting with a squared error function, which in addition to improving the accuracy of the model assists in the selection of the sparse set of pixels in combination with the prior probability of the distances between pairs of pixels. The model presents a response on the order of milliseconds, being well suited for the application at hand. The training process used the 300-W dataset [38], which consists of 300 images obtained indoors and 300 in outdoor conditions with spontaneous expressions, all manually annotated. This dataset includes face images collected during several outdoor activities such as sports, parties, or protests, presenting large variations in both illumination conditions (including poor illumination) and facial expressions. Therefore, it is particularly useful for our application, given that it is required to robustly detect facial landmarks in different illumination conditions when driving.

After obtaining the facial landmarks, the proposed model focuses on the recognition of opening and closing of eyes and mouth. With this objective, markers for the right eye (38,39,41,42), left eye (44,45,47,48), and mouth (62–64,66–68) are tracked for computing the distance between eyelids and lips. Eyes are considered closed when the space between eyelids is less than 20% of the open eye area (as defined in [39]), and the mouth is considered opened when its aspect ratio (mar=Height/Width) is greater than 0.7, as defined in [10].

Given the different characteristics of subjects’ facial features (eyes and mouth) and opening area, the developed system includes a setup step of at least one minute in which the subject is monitored to determine a personalized threshold value of open eyes and mouth areas. When switching on the device, the driver is prompted to maintain their position in front of the camera while continuing with the driving activities (leaving their mouth open at least twice for a small period). Then, for each frame obtained during the selected time window (in our case the duration was defined as 1 min), the device measures the *mar* metric and the space between eyelids (at a 30 fps rate, 1800 values for each eye and *mar* are obtained). The average of 5% most significant distances (which indicates opened eyes and mouth) is employed to calculate the thresholds that determine eyes or mouth openings. Therefore, the system can adapt to different drivers without any variation in its accuracy for recognizing eyes closing or mouth opening.

### 2.3. Driver Drowsiness Indicators

The selected driver drowsiness indicators are based on the principle that a combination of complementary measures can produce a more robust system, overcoming the limitations of each one separately. The selected indicators are: PERCLOS [8], ECD [8], and AOT [10]. Each of those measures is computed based on the states obtained for eyes (PERCLOS, ECD) and mouth (AOT).

PERCLOS is a prevalent indicator of drowsiness in behavioral-measures-based systems. It is defined as the proportion of frames in which the driver has their eyes closed in a determined temporal window [8]:

(1) $$PERCLOS\left[i\right]=\frac{\sum_{j=i-n+1}^{i}frame\left[j\right]}{n}$$

where PERCLOS[i] indicates the value of PERCLOS for the selected window at instant *i*, n is the number of frames obtained in the temporal window, and frame[j] is a binary value that indicates if the eye was closed in *frame j.*

ECD is defined as the mean duration of intervals in which eyes are closed in a determined temporal window:

(2) $$ECD\left[i\right]=\frac{\sum_{j=1}^{p}Duration\left[j\right]}{p}$$

where ECD[i] represents the values of ECD at time *i*, p is the number of intervals in a defined temporal window, and Duration[j] is the duration of the lapse between closing and reopening the eyes.

AOT is defined as the mean duration of intervals in which the subject kept their mouth open during a specific time window. It can be expressed as:

(3) $$AOT\left[i\right]=\frac{1}{N_{y}}\overset{N_{y}}{\underset{j=1}{\sum }}t_{j}$$

where AOT[i] defines the AOT magnitude at instant *i*, $N_{y}$ is the number of yawns during the time window, and $t_{j}$ is the duration of yawn *j*. Yawning is considered as the action of keeping the mouth open for more than three seconds [10]. In our application, the yawning duration value begins after those three seconds.

These indicators are complementary, in the sense that if one of them gives a false positive, the others act as validators of the drowsiness condition. Moreover, the combination of PERCLOS and ECD strengthens the robustness of the system to variations in blinking frequency and eye state misclassifications [8].

### 2.4. Fuzzy Inference System Applied to Drowsiness Level Estimation

The combination of the three drowsiness indicators is performed by a Mamdani Fuzzy Inference System [40]. Therefore, the proposed Drowsiness Level Estimation module provides a nonlinear and interpretable mapping from the input indicators (PERCLOS, ECD and AOT) to the drowsiness level output (Low-Normal State, Medium-Drowsy State, and High-Severe Drowsiness State). The three indicators are calculated based on a temporal window size of one minute, computed each second. 

One essential aspect of the proposed system’s design is the specification of the limits that determine if a person is drowsy or not. In the literature, there is no consensus about these values for each of the metrics, defining the drowsiness threshold based on experimental results without any clinical validation. Table 1 presents, as an example, the different drowsiness thresholds for the PERCLOS indicator. This disagreement among experts makes the use of a fuzzy inference system even more critical, as FIS is known for being able to deal with these uncertainties. The values of the fuzzy sets corresponding to each variable were set considering the selected window size, experimental results, and reference values in the literature.

**Figure 3:**
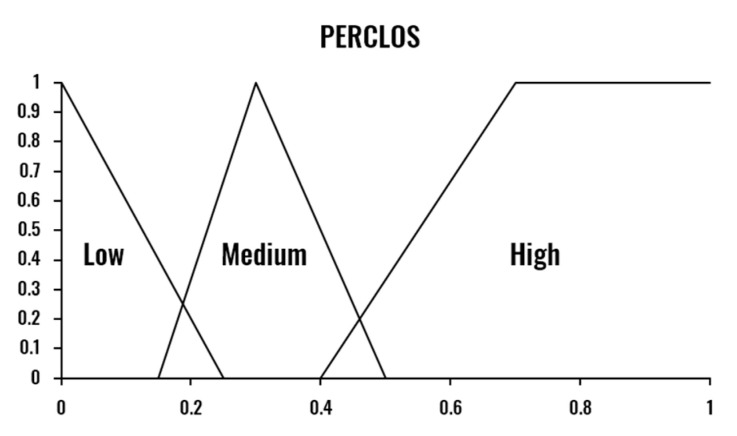
Fuzzy membership functions for PERCLOS.

**Figure 4:**
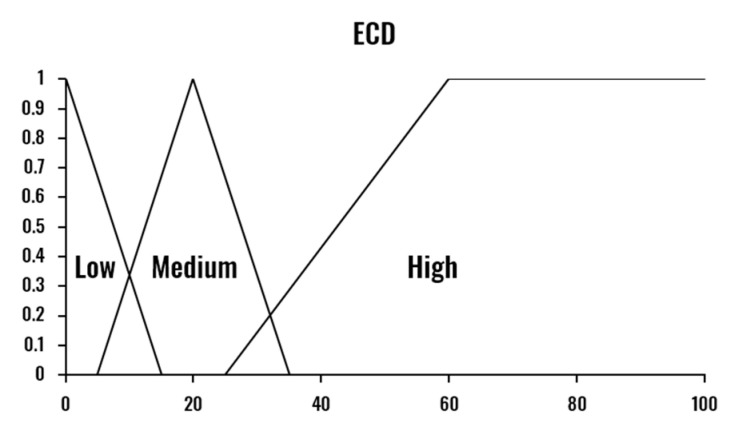
Fuzzy membership functions for eye closing duration (ECD).

**Figure 5:**
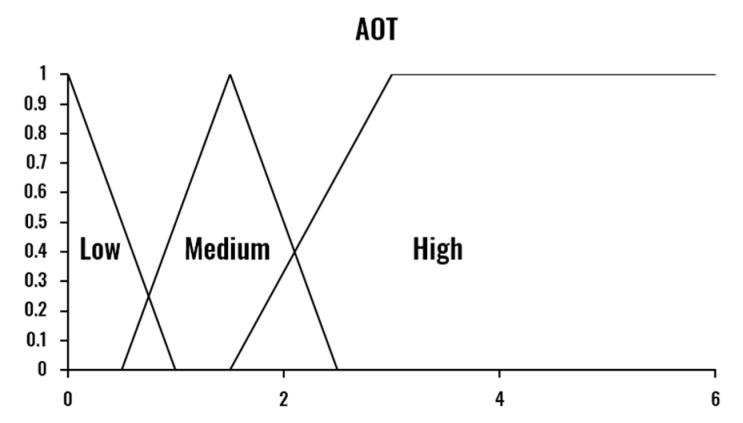
Fuzzy membership functions for average mouth opening time (AOT).

**Figure 6:**
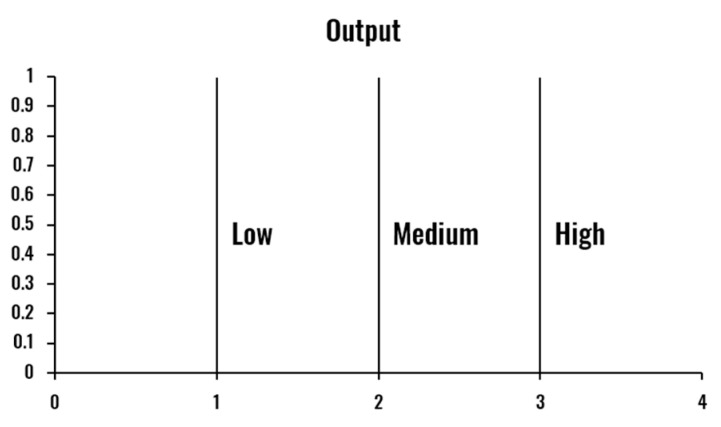
Fuzzy membership functions for drowsiness level output.

**Figure 7:**
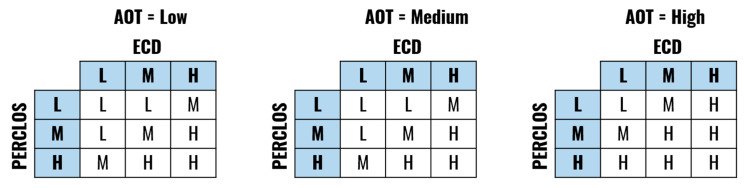
Fuzzy rules (L = Low, M = Medium, H = High).

The developed FIS is defined by the membership functions shown in Figure 3, Figure 4, Figure 5 and Figure 6 and a set of rules illustrated in Figure 7. The three output drowsiness levels (Low-Normal State, Medium-Drowsy State, and High-Severe Drowsiness State) are represented by singleton fuzzy sets (Figure 6). The rules were defined following the principle that the selected indicators (PERCLOS, ECD, and AOT) complement each other, allowing the system to reduce the effect of individual false positives and eye state misclassifications. Therefore, a Medium drowsiness level is produced by two eye states indicators increasing their level to medium, one of them increasing its level to high, or a combination of AOT at a high level and any of the other indicators at medium. Particularly, AOT influences an increment in the output drowsiness level when its level is high due to undesired effects on the metric produced by driver activities such as conversation or singing.

The fuzzy operator employed for AND connective and the Mamdani implication was the minimum; the maximum was chosen as the aggregation operator. The minimum of maximum was selected as the defuzzification method to provide the final classification output, given that the output membership functions are singletons.

### 2.5. Hardware

The main hardware component is the Raspberry Pi 3 Model B [43], which possesses a Quad Core 1.2 GHz Broadcom BCM2837 64 bit processor combined with a memory of 1 GB. The device operates as a minicomputer that executes the operating system Raspbian [44] (an operating system based on Debian and optimized for that hardware) that allows the developed software to be integrated in Python language and processes each frame with the other components. The device relies on a 5 V power source with 3 A, and an SD card with 16 GB of storage capacity to run the operating system.

Infrared (IR)-sensitive cameras, combined with proper external IR illumination, can generate excellent images for environments with little or no lightning and eliminate reflections caused by drivers’ glasses [45]. Therefore, this type of camera was selected to produce a system robust to ambient lightning variations and vision obstruction. The camera utilized in our device is the NoIR v2 of 8 MP, built for the Raspberry Pi hardware, combined with an external IR illumination of 48 LEDs that require a power source of 12 V and 1 A. The camera and IR illumination system are designed to be located on the dashboard above the steering wheel, with a direct vision of the driver.

## 3. Results and Discussion

The proposed model was evaluated through two different analyses. The first investigation was directed to the examination of the processing capacity of the portable hardware to analyze real-time images and the effects of continuous work on the device’s response. The second analysis focused on the determination of the performance obtained by the proposed model in recognizing different drowsiness states. In both cases, the portable device’s results were compared with those of a processing model deployed in a laptop, offering a benchmark comparison of the response of the model in a more powerful hardware setup. In this way, it was possible to compare how the drowsiness recognition accuracy of the model was affected by variations in the hardware computing capacity.

The laptop was equipped with an integrated webcam model Camera L 80WK HD 720P, an Intel Core I7-7700HQ processor, and 16 GB of RAM. The metrics were computed each second, employing samples with a one-minute window size. In each hardware setup, the model was developed in Python 3.6, running in the Anaconda environment on the Microsoft Windows 10 Operating System for the laptop and in the Raspbian Operating System for the Raspberry Pi.

### 3.1. Computational Performance of the Model in Real-Time

**Figure 8:**
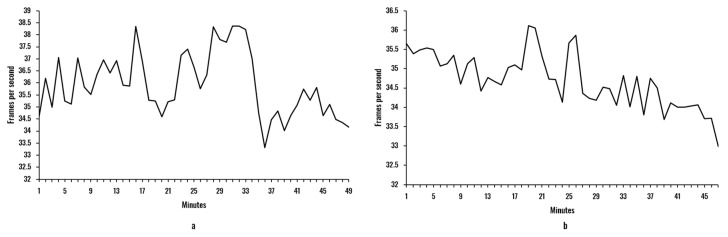
Mean of the fps per minute for the laptop hardware: (**a**) Haar model; (**b**) HOG model.

**Figure 9:**
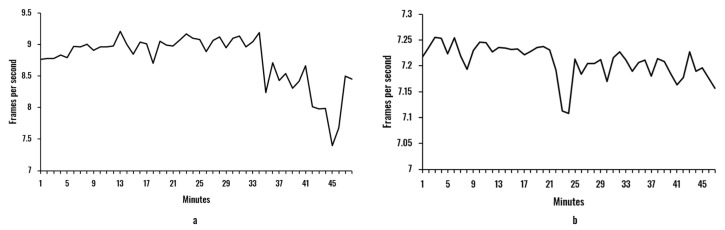
Mean of the fps per minute for the Raspberry Pi hardware: (**a**) Haar model; (**b**) HOG model.

The objective of this analysis was to verify the computational performance offered by the proposed model in combination with the portable hardware. Additionally, we wanted to compare this response with the one produced by a standard hardware setup (a laptop). We also wished to assess the effects of continuous working time on the processing capacity of the device. The evaluation was conducted using ten sample videos of approximately 50 min of users during work activities in front of the camera. Figure 8 and Figure 9 illustrate the mean frames per second of the two face recognition models and the two hardware setups.

The difference between hardware setups is evident when comparing Figure 8 and Figure 9. On the one hand, the laptop configuration was able to maintain the average number of processed frames per second over 32 for both facial recognition models. On the other hand, the Raspberry Pi was limited to values between 7–9.5 and 7–7.3 for the Haar-features-based and HOG models, respectively. This remarkable difference is a result of the massive computing power demanded by those facial recognition models (the most time-consuming stage of the processing model). Another effect observed is a small tendency (in all cases) to decrease the fps with time. Although a more profound analysis should be implemented to derive more accurate conclusions related to this issue, it can be stated that the continuous processing imposes a limit on the processing capacity, with probable overheating in both laptop and Raspberry Pi devices.

When comparing the Haar and HOG models, it can be seen that for both hardware setups, the HOG model presented a reduction in the fps values, with fewer variations than the fps of the Haar model (see Table 2). Specifically, the Haar model offered an average of 35.93 and 8.75 fps for laptop and Raspberry Pi setups, respectively, with standard deviations of 1.31 and 0.59. Remarkably, the Raspberry Pi setup, even with a much slower response, presented less variability in the fps rate, reducing a standard deviation of 0.72. The HOG model resulted in smaller values, with 34.70 fps for the laptop and 7.21 fps for the Raspberry Pi. The standard deviation, on the other hand, was lower when compared to the Haar models, with 0.70 and 0.07 for both hardware setups. Particularly, during the experiments, it was perceived that for the Haar model, face rotation of more than 30° resulted in instability in the face recognition, while the HOG model was more robust in this aspect.

To improve the fps rate in the Raspberry Pi (our proposed portable model), we explored a computing performance improvement strategy based on the experimental evidence that the facial recognition is the stage in which the algorithms take a longer time. Therefore, the size of the image employed in the facial recognition stage was reduced, maintaining the aspect ratio. After obtaining the coordinates of the region of the smaller image in which the face is situated, these values were resized to represent the equivalent region in the original image resolution (area of the original image that contains the face). This strategy produced the improvements in computing performance described in Table 3. As expected, rescaling the image had an inversely proportional influence on the fps rate of the system, with steady increments when reducing the reduction factor until doubling the performance (15.94 fps for the Haar model and 14.77 for the HOG model) for a reduction factor of 0.5. The standard deviation generally presented a small increment for both models, indicating that smaller images produced more pronounced variations of the fps.

These results confirm our conclusions related to the face recognition models. In all cases, the mean fps of the Haar model was superior to the mean fps of the HOG model by around 1 fps, but with a more significant standard deviation. By reducing the image to half its original size, the performance doubled.

### 3.2. Accuracy of the Drowsiness Recognition System

To evaluate the accuracy of the proposed model, four acquisition experiments were designed based on video streams of approximately 50 min from two subjects (two videos by subject) of 24 and 28 years old, each without preexisting medical conditions. The recorded videos were scripted and contained simulations of different combinations of eyes and mouth states that defined different drowsiness conditions. The acquisition setup emulated the position of the subject and the inclination of the camera when installed in a car. Given those specific conditions, the simulations can be compared to the real drowsiness level provided by the fuzzy inference system, producing an assessment of the accuracy of the proposed device. The video resolution was set to 640 × 480. Table 4 summarizes the total number of different drowsiness states included in the experiments, divided into segments with a size of approximately 1 min.

**Figure 10:**
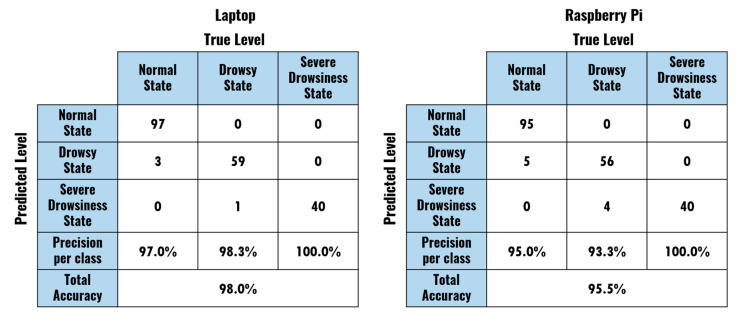
Confusion matrices describing the performance of the model.

The accuracy of the model was evaluated by comparing the subject’s state in each moment (segments of the experiment in which the subject simulated drowsiness symptoms) with the FIS output. The results of this assessment for the laptop- and Raspberry-Pi-based models are presented in Figure 10.

When analyzing the results of the proposed model in the portable hardware configuration, it can be stated that the proposed system presented a high accuracy (95.5%). Remarkably, the recognition errors were produced by misclassifications of Normal State segments as Drowsy State segments (five for the Raspberry Pi and three for the laptop) and Drowsy State segments as Severe Drowsiness State segments (four for the Raspberry Pi and one for the laptop). Although these errors indicate some limitations of the proposed system in some specific segments, they are not as relevant as a misclassification of drowsiness states into the normal state (not present in our model), which would fail the purpose of the device (to alarm the subject when tired). Additionally, the high accuracy obtained for the two subjects in the four experiments confirms the relevance of the adaptation stage included in the system setup. Through this process, different threshold values were defined for each test when determining eyes closure and mouth opening, adjusting the device to different conditions.

There was a 2.5% difference between the results of the laptop and the Raspberry Pi in terms of total accuracy. Notably, the laptop-based model recognized both Normal and Drowsy States with higher accuracy. Both approaches were able to accurately identify the segments representing Severe Drowsiness. Those results indicate that the differences in frames processed per second (see Section 3.1) did not produce a significant difference in the performance of the model.

**Figure 11:**
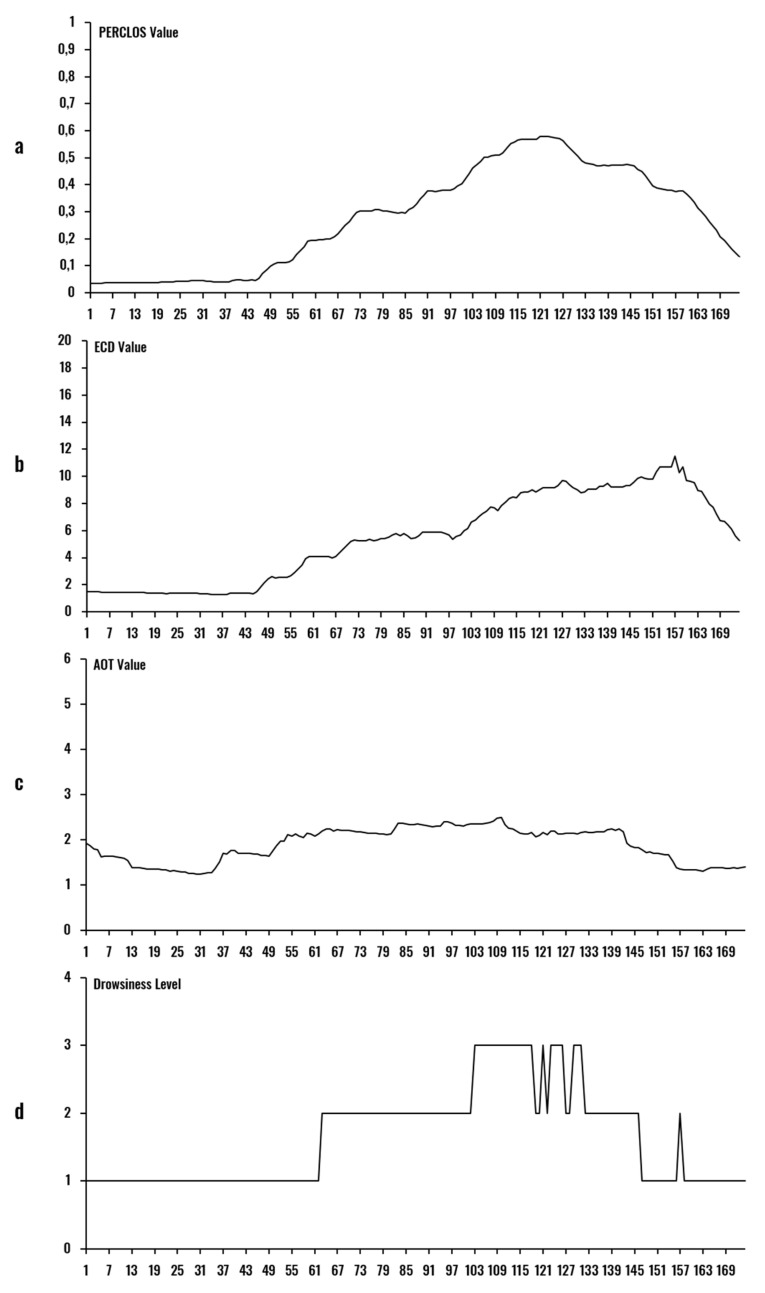
Example of output of the model for 175 s processing: (**a**) PERCLOS; (**b**) ECD; (**c**) AOT; (**d**) Drowsiness level.

An example of the output of the portable model for a given user is illustrated in Figure 11. Items (a), (b), and (c) are the magnitudes of PERCLOS, ECD, and AOT per second, respectively. The graph represented in (d) is the subject’s estimated Drowsiness Level. The time segment begins with the driver in an alert state, transitioning to a simulated drowsy state after the 40th second.

Note that in our proposed system we are monitoring only facial features, which might prevent the accurate recognition of the drowsiness state in some specific situations that can generate total facial occlusion or head-turning. Additionally, cases where the driver presents a behavior deviating from their usual one when driving (e.g., an extremely high number of blinks in a short time or frequently opening their mouth) can generate imprecisions in the recognition. Nevertheless, as described in Section 2.3 and Section 2.4, the selected facial features are complementary, and the FIS is designed to avoid false-positive drowsiness detection from only one of the measures. To generate a more robust system based on behavioral measures, one can add the detection of head inclination and turning.

#### Extended Performance Validation

**Figure 12:**
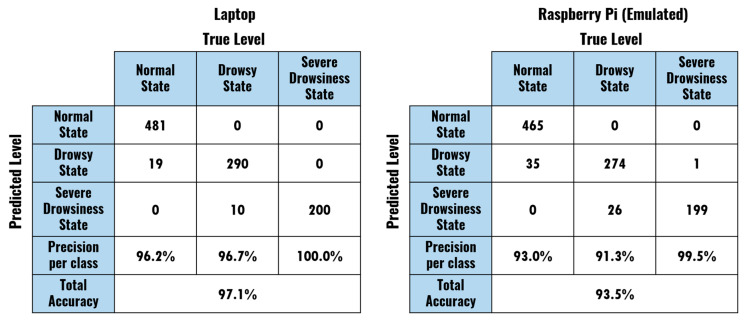
Confusion matrices describing the performance of the model for a more significant number of subjects.

To extend the analysis of the system’s performance and to validate its adaptability to different users, new tests were performed over a more significant set of subjects, with a broader range of ages and genders, following the same protocol (50 min duration). Eight new subjects were included, with six males and four females in total: two in the range of 20 to 30 years; one in the range of 30 to 40 years; one in the range from 40 to 50 years; two in the range from 50 to 60; and two in the range of 70 to 80 years. The tests were applied using laptops, but emulating the limitations of the Raspberry Pi setup by eliminating frames from the stream and designing the system to process images only in the corresponding time instants (frames per second reduced to 14). The resulting quantities of ~1-min segments from these experiments were: 500 of Normal State, 300 of Drowsy State, and 200 of Severe Drowsiness State. The obtained accuracy of the system in this extended dataset is shown in Figure 12.

When applied on a larger number of subjects, the performance of the model dropped slightly for the laptop setup (from 98.0% total accuracy to 97.1%) and more significantly for the emulated Raspberry Pi setup (from 95.5% total accuracy to 93.5%). In general, the tendency of the model was maintained when misclassifying examples, mistakenly assigning Drowsy State labels to Normal State ones (19 samples for the laptop and 35 for the emulated Raspberry Pi), and Severe Drowsiness State labels to Drowsy State examples (10 and 26 cases for the laptop and emulated Raspberry Pi, respectively). For both setups, an outstanding performance for Severe Drowsiness State recognition was observed, with only one example misclassified as Drowsy State for the emulated Raspberry Pi. These preliminary results validate the model’s capacity to accurately recognize the drowsiness level of subjects of different ages in laboratory conditions. Extensive tests should be conducted to validate the model in real driving situations, including even more subjects.

## 4. Conclusions

This paper introduced the development of a low-cost portable device that assesses the drowsiness level of a driver. The device is based on an IR illuminator and a camera that streams the image acquired to a Raspberry Pi 3 Model B, resulting in a device which is robust to variations in the ambient lighting condition and vision obstruction. The processing model combines measures obtained from the eyes and mouth (PERCLOS, ECD, and AOT) and provides the drowsiness level as the output of a fuzzy inference system (Low-Normal State, Medium-Drowsy State, and High-Severe Drowsiness State). Two different facial features recognition methods were evaluated: one that employed a cascade classifier and Haar-like filter features and another that applied a linear support vector machine SVM with HOG features.

The proposed device was tested in four experiments comprising two subjects in different simulated drowsiness conditions with approximately 50 min duration. The average accuracy of the system in recognizing different drowsiness states was 95.5%. The processing model was also implemented in a laptop to compare the effects of reducing the computational power (using the Raspberry Pi) for the portable hardware. The difference in the recognition accuracy was only 2.5%, even with a difference in the mean number of frames processed by the hardware of approximately 17 frames. Additional tests were implemented with more subjects, covering a broader age range, resulting in a significant accuracy of 97.1% and 93.5% for the laptop and emulated Raspberry Pi based models, respectively.

The main advantages of the proposed device are: adaptability to different drivers’ facial features, low cost, robustness to changes in ambient illumination and vision occlusion, and freedom in the design (allowing the device owner to modify the fuzzy membership functions domains and temporal window for the calculation of PERCLOS, ECD, and AOT). This last feature allows the adjustment of the device to specific conditions desired for the application, such as a faster response of the output (by decreasing the temporal window size) or new drowsiness indications (modifying the limits of the fuzzy membership functions).

As future work, the model should be evaluated on real driving conditions and a more significant number of subjects in order to validate the conclusions obtained in this work on a practical application. Additionally, we intend to investigate the enhancement of the fps obtained from the portable model (the fps obtained by the Raspberry Pi was far from the fps rate achieved with the laptop-based setup). This improvement can be explored by different approaches. In the first case, more powerful portable hardware devices can be evaluated, like the Raspberry Pi 4, Nvidia Jetson Nano, Asus Tinker Board, or Rock64 embedded systems. Secondly, the possibility of coupling the Raspberry Pi with a neural stick such as the Intel Neural Compute Stick 2 could be explored, which, for a medium increment in the price, would make it possible to explore deep-learning-based image processing in real time. We also intend to set up the proposed model in real vehicles to assess the effects of actual driving conditions on its operation. Given the lack of information in the literature about specific thresholds for the drowsiness measures (PERCLOS, ECD, AOT), and the divergences between the research that reported such information, it would be interesting to conduct a broader investigation that helps to unify the decision criteria. Additionally, the monitoring of head inclination and turning in the system could be included, which could enhance the system’s robustness by adding a measure not limited to facial features without modifying the proposed hardware.

## Figures and Tables

**Table 1:** Drowsiness thresholds reported in the literature for percentage of eyelid closure (PERCLOS).

Drowsiness Threshold	Window Size	FPS	Reference
0.02	1, 2 min	25	[32]
0.3, 0.25		4–5	[41]
0.2		25	[42]
0.2	8, 10 s	25	[8]
0.4	24–25 s	25	[12]
0.15, 0.18	30 s	7	[9]

**Table 2:** Real-time response of face detection models in different hardware.

Model	Haar		HOG	
Hardware Setup	Mean fps	Standard Deviation	Mean fps	Standard Deviation
Laptop	35.93	1.31	34.70	0.70
Raspberry Pi	8.75	0.59	7.21	0.07

**Table 3:** Performance obtained with fps enhancement strategies.

Model	Haar		HOG	
Reduction Factor	Mean fps	Standard Deviation	Mean fps	Standard Deviation
Original	8.75	0.59	7.21	0.07
0.7	10.58	0.52	9.6	0.11
0.6	12.14	0.7	11.17	0.16
0.5	15.94	0.66	14.77	0.29

**Table 4:** Testing drowsiness data.

Drowsiness Level	Segments of Approximately 1 min Duration
Normal State	100
Drowsy State	60
Severe Drowsiness State	40

## References

[1] Owens J.M., Dingus T.A., Guo F., Fang Y., Perez M., McClafferty J., Tefft B. (2018). Prevalence of Drowsy-Driving Crashes: Estimates from a Large-Scale Naturalistic Driving Study.

[2] Veruska Narciso F.I., Túlio de Mello M.I., Túlio de Mello M. (2017). Segurança e saúde dos motoristas profissionais que trafegam nas rodovias do Brasil. Rev. Saude Publica.

[3] Higgins J.S., Michael J., Austin R., Åkerstedt T., Van Dongen H.P., Watson N., Czeisler C., Pack A.I., Rosekind M.R. (2017). Asleep at the wheel-the road to addressing drowsy driving. Sleep.

[4] Sahayadhas A., Sundaraj K., Murugappan M. (2012). Detecting driver drowsiness based on sensors: A review. Sensors.

[5] Kaplan S., Guvensan M.A., Yavuz A.G., Karalurt Y. (2015). Driver behavior analysis for safe driving: A survey. IEEE Trans. Intell. Transp. Syst..

[6] Wang Q., Yang J., Ren M., Zheng Y. (2006). Driver fatigue detection: A survey. Proc. World Congr. Intell. Control Autom..

[7] Ramos A.C., Vellasco M. (2018). Quantum-inspired evolutionary algorithm for feature selection in motor imagery EEG classification. Proceedings of the 2018 IEEE Congress on Evolutionary Computation, CEC 2018.

[8] Jo J., Lee S.J., Park K.R., Kim I.J., Kim J. (2014). Detecting driver drowsiness using feature-level fusion and user-specific classification. Expert Syst. Appl..

[9] Zheng R., Tian C., Li H., Li M., Wei W. (2015). Fatigue detection based on fast facial feature analysis. Proceedings of the Lecture Notes in Computer Science (Including Subseries Lecture Notes in Artificial Intelligence and Lecture Notes in Bioinformatics).

[10] Zhu W., Yang H., Jin Y., Liu B. (2017). A method for recognizing fatigue driving based on dempster-shafer theory and fuzzy neural network. Math. Probl. Eng..

[11] Lin L., Huang C., Ni X., Wang J., Zhang H., Li X., Qian Z. (2015). Driver fatigue detection based on eye state. Technol. Heal. Care.

[12] Zhang C., Wei L., Zheng P. (2017). Research on driving fatigue detection based on PERCLOS. Proceedings of the 4th International Conference on Vehicle, Mechanical and Electrical Engineering, ICVMEE 2017.

[13] Abulkhair M., Alsahli A.H., Taleb K.M., Bahran A.M., Alzahrani F.M., Alzahrani H.A., Ibrahim L.F. (2015). Mobile platform detect and alerts system for driver fatigue. Procedia Comput. Sci..

[14] Kong W., Zhou L., Wang Y., Zhang J., Liu J., Gao S. (2015). A system of driving fatigue detection based on machine vision and its application on smart device. J. Sens..

[15] Manoharan R., Chandrakala S. (2015). Android OpenCV based effective driver fatigue and distraction monitoring system. Proceedings of the International Conference on Computing and Communications Technologies, ICCCT 2015.

[16] Jabbar R., Al-Khalifa K., Kharbeche M., Alhajyaseen W., Jafari M., Jiang S. Real-time driver drowsiness detection for android application using deep neural networks techniques. Proceedings of the 9th International Conference on Ambient Systems, Networks and Technologies.

[17] Galarza E.E., Egas F.D., Silva F.M., Velasco P.M., Galarza E.D. Real time driver drowsiness detection based on driver’s face image behavior using a system of human computer interaction implemented in a smartphone. Proceedings of the International Conference on Information Technology & Systems.

[18] Wijnands J.S., Thompson J., Nice K.A., Aschwanden G.D.P.A., Stevenson M. (2019). Real-time monitoring of driver drowsiness on mobile platforms using 3D neural networks. Neural Comput. Appl..

[19] Fei W., Huabiao Q. A FPGA based driver drowsiness detecting system. Proceedings of the 2005 IEEE International Conference on Vehicular Electronics and Safety, ICVES 2005.

[20] Li Z., Zhang F., Sun G., Zhao D., Zheng K. Driver fatigue detection system based on DSP platform. Proceedings of the 22nd International Conference on Multimedia Modeling.

[21] Selvakumar K., Jerome J., Rajamani K., Shankar N. (2016). Real-time vision based driver drowsiness detection using partial least squares analysis. J. Signal Process. Syst..

[22] Teach, Learn, and Make with Raspberry Pi–Raspberry Pi. https://www.raspberrypi.org/.

[23] The Power of Modern AI to Millions of Devices | NVIDIA Jetson Nano. https://www.nvidia.com/en-us/autonomous-machines/embedded-systems/jetson-nano/.

[24] Tinker Board | Single Board Computer | ASUS Global. https://www.asus.com/Single-Board-Computer/Tinker-Board/.

[25] ROCK64 | PINE64. https://www.pine64.org/devices/single-board-computers/rock64/.

[26] Mavely A.G., Judith J.E., Sahal P.A., Kuruvilla S.A. Eye gaze tracking based driver monitoring system. Proceedings of the IEEE International Conference on Circuits and Systems.

[27] Mena D.M.L., Rosero A.F.C. Portable artificial vision system to determine fatigue in a person using a raspberry PI3 card. Proceedings of the 2017 International Conference on Information Systems and Computer Science.

[28] Hossain M.Y., George F.P. IOT based real-time drowsy driving detection system for the prevention of road accidents. Proceedings of the 2018 International Conference on Intelligent Informatics and Biomedical Sciences.

[29] Ghazal M., Abu Haeyeh Y., Abed A., Ghazal S. Embedded fatigue detection using convolutional neural networks with mobile integration. Proceedings of the IEEE 6th International Conference on Future Internet of Things and Cloud Workshops.

[30] Kim W., Jung W.-S., Choi H.K. (2019). Lightweight driver monitoring system based on multi-task mobilenets. Sensors.

[31] Wierwille W.W., Wreggit S.S., Kirn C.L., Ellsworth L.A., Fairbanks R.J. (1994). Research on Vehicle-Based Driver Status/Performance Monitoring; Development, Validation, and Refinement of Algorithms for Detection of Driver Drowsiness.

[32] Sigari M.H., Fathy M., Soryani M. (2013). A driver face monitoring system for fatigue and distraction detection. Int. J. Veh. Technol..

[33] Rigane O., Abbes K., Abdelmoula C., Masmoudi M. A fuzzy based method for driver drowsiness detection. Proceedings of the IEEE/ACS 14th International Conference on Computer Systems and Applications, AICCSA.

[34] Viola P., Jones M. Rapid object detection using a boosted cascade of simple features. Proceedings of the IEEE Computer Society Conference on Computer Vision and Pattern Recognition.

[35] Dalal N., Triggs B. Histograms of oriented gradients for human detection. Proceedings of the 2005 IEEE Computer Society Conference on Computer Vision and Pattern Recognition, CVPR 2005.

[36] Schapire R.E., Schoelkopf B., Luo Z., Vovk V. (2013). Explaining adaboost. Empirical Inference: Festschrift in Honor of Vladimir, N. Vapnik.

[37] Kazemi V., Sullivan J. (2014). One millisecond face alignment with an ensemble of regression trees. Proceedings of the IEEE Computer Society Conference on Computer Vision and Pattern Recognition.

[38] Sagonas C., Tzimiropoulos G., Zafeiriou S., Pantic M. 300 faces in-the-wild challenge: The first facial landmark Localization Challenge. Proceedings of the 2013 IEEE International Conference on Computer Vision.

[39] Lin L., Huang C., Ni X., Wang J., Zhang H., Li X., Qian Z. Driver fatigue detection based on eye state. Proceedings of the 3rd International Conference on Biomedical Engineering and Technology.

[40] Mamdani E.H., Assilian S. (1975). An experiment in linguistic synthesis with a fuzzy logic controller. Int. J. Man. Mach. Stud..

[41] Flores M.J., Armingol M.J.M., de la Escalera A. (2011). Sistema avanzado de asistencia a la conducción para la detección de la somnolencia. Rev. Iberoam. Autom. Inform. Ind. RIAI.

[42] Jo J. (2011). Vision-based method for detecting driver drowsiness and distraction in driver monitoring system. Opt. Eng..

[43] Buy a Raspberry Pi 3 Model B–Raspberry Pi. https://www.raspberrypi.org/products/raspberry-pi-3-model-b/.

[44] Front Page-Raspbian. https://www.raspbian.org/.

[45] Shoja Ghiass R., Arandjelović O., Bendada A., Maldague X. (2014). Infrared face recognition: A comprehensive review of methodologies and databases. Pattern Recognit..

